# Identification of Direct Target Engagement Biomarkers for Kinase-Targeted Therapeutics

**DOI:** 10.1371/journal.pone.0026459

**Published:** 2011-10-24

**Authors:** Cloud P. Paweletz, Jannik N. Andersen, Roy Pollock, Kumiko Nagashima, Mansuo L. Hayashi, Shangshuan U. Yu, Hongbo Guo, Ekaterina V. Bobkova, Zangwei Xu, Alan Northrup, Peter Blume-Jensen, Ronald C. Hendrickson, An Chi

**Affiliations:** 1 Merck & Co., Inc., Boston, Massachusetts, United States of America; 2 Merck & Co., Inc., West Point, Pennsylvania, United States of America; 3 Metamark Genetics, Inc, Cambridge, Massachusettes, United States of America; 4 Merck & Co., Inc., Rahway, New Jersey, United States of America; George Mason University, United States of America

## Abstract

Pharmacodynamic (PD) biomarkers are an increasingly valuable tool for decision-making and prioritization of lead compounds during preclinical and clinical studies as they link drug-target inhibition in cells with biological activity. They are of particular importance for novel, first-in-class mechanisms, where the ability of a targeted therapeutic to impact disease outcome is often unknown. By definition, proximal PD biomarkers aim to measure the interaction of a drug with its biological target. For kinase drug discovery, protein substrate phosphorylation sites represent candidate PD biomarkers. However, substrate phosphorylation is often controlled by input from multiple converging pathways complicating assessment of how potently a small molecule drug hits its target based on substrate phoshorylation measurements alone. Here, we report the use of quantitative, differential mass-spectrometry to identify and monitor novel drug-regulated phosphorylation sites on target kinases. Autophosphorylation sites constitute clinically validated biomarkers for select protein tyrosine kinase inhibitors. The present study extends this principle to phosphorylation sites in serine/threonine kinases looking beyond the T-loop autophosphorylation site. Specifically, for the 3′-phosphoinositide-dependent protein kinase 1 (PDK1), two phospho-residues p-PDK1^Ser410^ and p-PDK1^Thr513^ are modulated by small-molecule PDK1 inhibitors, and their degree of dephosphorylation correlates with inhibitor potency. We note that classical, ATP-competitive PDK1 inhibitors do not modulate PDK1 T-loop phosphorylation (p-PDK1^Ser241^), highlighting the value of an unbiased approach to identify drug target-regulated phosphorylation sites as these are complementary to pathway PD biomarkers. Finally, we extend our analysis to another protein Ser/Thr kinase, highlighting a broader utility of our approach for identification of kinase drug-target engagement biomarkers.

## Introduction

Drug development remains an expensive, uncertain and challenging endeavor. The cost from discovery to regulatory approval of a novel drug averages about $900 million out of pocket, with a capitalized cost of ∼$1.78 billion, and the process now typically takes up towards 13 years [1]–[3]. Moreover, on average nine out of ten drugs entering clinical development do not receive marketing approval and projects often fail to provide proof-of-concept for efficacy (Ph II) or in late development (Phase III) adding a high degree of risk to drug development [1], [2]. Consequently, the pharmaceutical industry is looking for ways to improve the probability of success for each drug development candidate by selecting not only the best compounds, but also drug-specific biomarkers to precisely measure the level of target inhibition and identify the patients likely to benefit from the drug as early as possible [4]. In fact, the incorporation of biomarkers early in the drug discovery process has become of such importance that it informs ‘go/no-go’ decisions on moving drug candidates forward towards the clinic [5]–[10].

Biomarkers are defined as molecular entities that can be measured and evaluated objectively as indicators of normal biological processes, pathogenic processes or pharmacological response to therapeutics and can be categorized as either proximal (target-related) or distal (disease-related) [11], [12]. Some of the guiding principles underlying biomarker-driven drug discovery and development have been conceptualized through a series of sequential questions: *Are there active concentrations of drug in plasma, blood and tissue? Is the target engaged? Is there downstream pathway inhibition? Is there a biological effect or clinical response?* [10]. While pharmacokinetic (PK) measurements of drug concentrations in plasma are routine, establishing whether a drug interacts with its molecular target (i.e. inhibition/activation) sufficiently to obtain a biological response has proven to be a challenging endeavor.

For kinase drug discovery, phosphorylated protein substrates of a kinase drug target represent attractive PD biomarker candidates. However, an inverse linear relationship between the stoicheometry of substrate phosphorylation and the degree of kinase inhibition seldom exists as the phosphorylation state of a given protein usually depends on convergent upstream input from multiple signaling pathways as well as negative and positive feedback loop regulation. Moreover, the phosphorylation of a particular substrate site is subject to variable rates of turnover depending on the dedicated phosphatase activity against said phosphorylation site. Despite these limitations, if a drug with a novel unproven mechanism fails to achieve the desired clinical activity, evaluation of PD biomarkers, when available, is an important means to assess the degree of target engagement by the drug. If the PD biomarkers indicate strong target engagement, the lack of clinical activity is likely due to the biology/mechanism of the target. Conversely, minimal target engagement due to non-favorable properties of the molecule or insufficient dosing will dictate additional pharmacological activities to address these issues.

For protein kinases, amino acid sequence alignments combined with site-directed mutagenesis and enzymatic assay have revealed conserved regulatory phosphorylation sites [13], [14]. For many kinases, a direct relationship between the phosphorylation state of such regulatory residues and their catalytic activity has been established [15]. Hence, monitoring the phosphorylation status of the activation site in kinase drug targets is a direct means of assessing drug-mediated kinase inhibition, and hence it serves as a direct PD biomarker. Specifically, for protein tyrosine kinases, monitoring the inhibition of kinase T-loop phosphorylation has been used to support the clinical development of a number of small-molecule inhibitors targeting EGFR, HER2 and cMET [16]–[18]. So far, this approach has been limited to a few protein tyrosine kinases with well-characterized autophosphorylation sites. Although activation of phosphorylation of Ser/Thr protein kinases is well studied in phosphorylation dependent signaling, it is also more complex, and subject to a multitude of allosteric regulatory mechanisms, via *cis* or *trans*- activation [19]–[21] rendering this approach challenging at best for this class of drug targets .

In order to assess kinase drug-target engagement independent of prior knowledge of kinase (auto)phosphorylation sites, such as the conserved T-loop phosphorylation site, we here report a broadly applicable, quantitative, differential mass spectrometric (MS) approach for identification and quantification of all direct and indirect drug-regulated phosphorylation sites in any kinase drug target.

We recently characterized a panel of structurally diverse PDK1-targeted lead compounds that inhibit the phosphorylation of PDK1 substrates such as AKT^Thr308^ and p90RSK^Ser221^ in multiple cell lines [22]. In that study, we also reported that treatment of cells with classical, ATP-competitive PDK1 kinase inhibitors do not result in decreased PDK1 T-loop autophosphorylation levels eliminating this phosphorylation site (p-PDK1^Ser241^) as a candidate target engagement biomarker for PDK1 inhibitor development. While it is informative to monitor the inhibition of proximal PD biomarkers (i.e. p-AKT^Thr308^ and p-p90RSK^Ser221^) in response to PDK1 inhibitor treatment, it is well established that the phosphorylation state of AKT is regulated indirectly by many other protein kinases including a majority of receptor protein tyrosine kinases, as well as lipid kinases, most notably phosphoinositide 3′-kinase (PI3′K). Accordingly, the observation that a PDK1-targeted small molecule inhibitor modulates p-AKT^Thr308^ does not guarantee that this is due to direct inhibition of PDK1, despite PDK1 is the upstream activating kinase of AKT that directly phosphorylates the T-loop phosphorylation site Thr308 in AKT. Based on these facts, we set out to identify drug-regulated phosphorylation sites on PDK1 that could be used to link direct PDK1 drug-target engagement in cells to biological responses. Specifically, we mapped and quantified 12 serine/threonine phosphorylation sites on PDK1, including novel phosphorylation sites, and found that two of these sites, p-PDK1^Ser410^ and p-PDK1^Thr513^, were modulated by pharmacological inhibitors of PDK1. Moreover, the degree of dephosphorylation of these sites correlated with the inhibitor potency (IC_50_) in PDK1 enzymatic assays.

## Results

**Figure 1:**
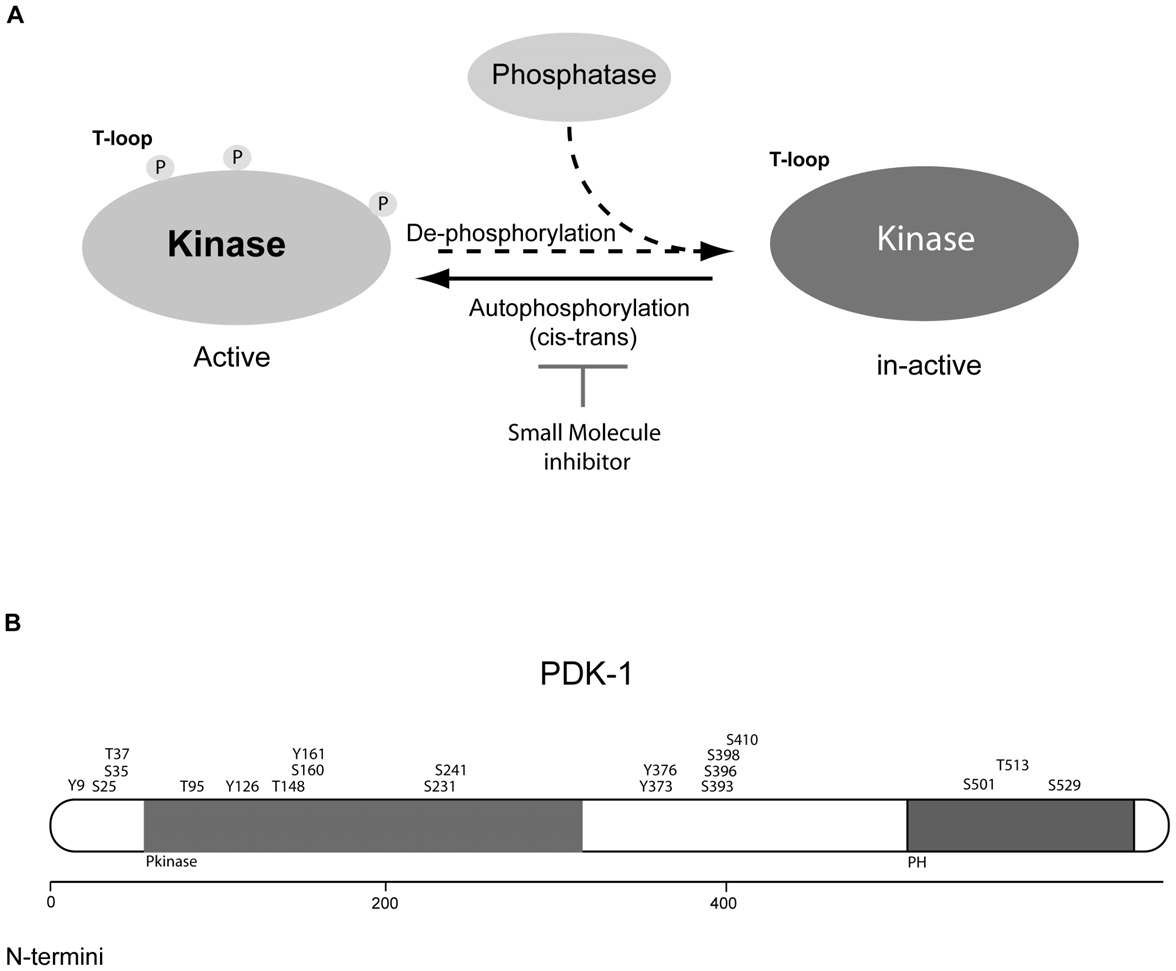
Large scale phosphorylation analyses add an unprecedented layer of information to kinases. **A**) Regulation of enzyme activity by reversible protein phosphorylation. Because the phosphorylation state of kinases often correlates with enzymatic activity, monitoring site-specific phosphorylation events are attractive biomarkers for measuring drug-target inhibition. **B**) Previously reported phosphorylation sites on PDK1.

The catalytic activity of protein kinases is tightly controlled by reversible protein phosphorylation [23], [24]. Protein autophosphorylation operates through a *cis* inhibition/*trans* activation mechanism and phosphorylation changes exerted by upstream kinases or phosphatases regulate kinase catalytic activity through allosteric conformational changes [18], [25], [26]. Under normal physiological conditions where kinase activity is tightly controlled, a unifying mechanism for oncogenic conversion, whether through point mutations, fusion events, or amplifications, is the relief of autoinhibitory constraints usually reflected in enhanced autophosphorylation [22]. Accordingly, drug-mediated inhibition of such (auto)phosphorylation sites provides a direct means for assessing kinase drug target engagement and inhibition as it often reports on the enzymatic activity of the kinase in cells (Figure 1A). Recent mass spectrometric studies have identified a vast number of novel protein phosphorylation sites in the human kinome as illustrated by the phosphorylation map for PDK1 (Figure 1B) [27]–[29]. However, regulatory functions for most identified phosphorylation sites remain poorly understood.

**Figure 2:**
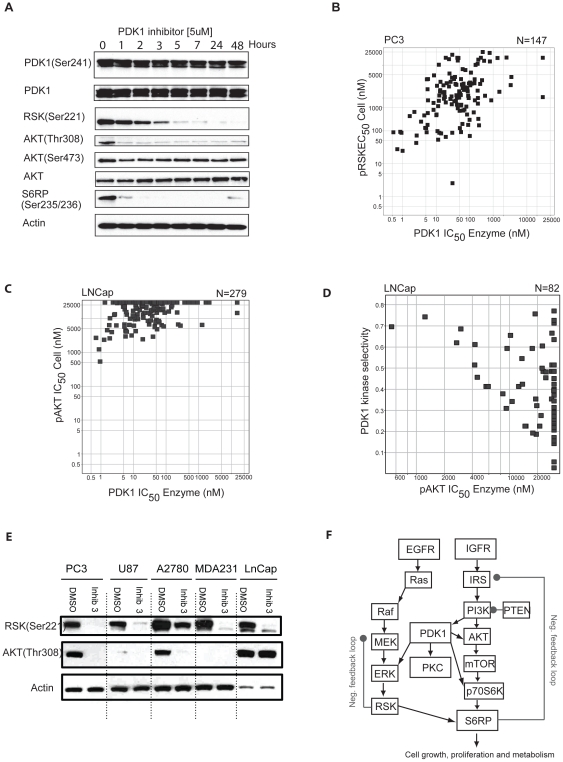
*Bona fide* substrates are not representative of PDK1 target inhibition. **A**) The T-loop phosphorylation site on PDK1^Ser241^ is not modulated by PDK1 inhibitor treatment in PC3 cells whereas downstream substrates, such as p-AKT^Thr308^ and p-RSK^Ser221^, are affected. **B**) In PC3 cells the inhibition of p-RSK^Ser221^ correlates with the enzymatic potency of PDK1 inhibitors (N = 147). **C**) LNCap cells don't exhibit a correlation between inhibition of p-AKT^Thr308^, a *bona fide* PDK1 substrate (N = 279), and the **D**) enzymatic potency of PDK1 inhibitors (N = 82). **E**) Western blot of p-AKT^Thr308^ and p-RSK^Ser221^ in a variety of cell lines. F) PDK1 substrates are subject to feedback regulation and pathway cross-talk.

We have recently characterized a panel of structurally diverse PDK1 inhibitors and found that classical, ATP-competitive inhibitors do not inhibit the PDK1 T-Loop phosphorylation site (Ser241) in PC3 cells, yet inhibit *bona fide* PDK1 substrates such as p-AKT^Thr308^ and p-RSK^Ser221^ [22] as shown in Figure 2A. Accordingly, to support the lead optimization of selective small-molecule PDK1 inhibitors, we established a Luminex® bead-based assay to measure p-AKT^Thr308^ inhibition in cells in response to inhibitor treatment (Figure 2B). The PTEN-deficient prostate cancer cell line LNCap was selected as a model system because of its high basal phosphorylation state of AKT and a robust assay performance using commercially available reference inhibitors of PI3K and AKT (data not shown). In parallel, an ‘in-cell’ western blotting assay was established [30] to measure the cellular inhibition of p-RSK^Ser221^ in PC3 cells (Figure 2C). In total, more than 400 compounds were tested in these assays and the cellular potency of compounds was correlated with their intrinsic activity against recombinant, purified PDK1 enzyme (Figure 2B & C) using the previously described enzymatic assays [30], [31]. In contrast to the observed correlation between the inhibitory potency against PDK1 enzymatic activity (IC_50_) and the cellular inhibition of p-RSK^Ser221^ in PC3 cells (Figure 2B), when small-molecule PDK1 inhibitors were tested in LNCap cells, the enzymatic potency of inhibitors against PDK1 did not correlate with the degree of inhibition of p-AKT^Thr308^ (Figure 2C). This is surprising, as p-AKT^Thr308^ is effectively inhibited in PC3 cells using a highly selective (>1000-fold) PDK1 inhibitor [22] and genetic knock-out of PDK1 completely abolishes the phosphorylation of AKT^Thr308^ in mouse embryonic fibroblasts (MEFs) [32]. Notably, kinase selectivity profiling of representative compounds showed that the inhibition of p-AKT^Thr308^ correlated with lack of kinome selectivity (Figure 2D). Thus, we conclude that the compound-mediated inhibition of p-AKT^Thr308^ in LNCap is likely due to off-target activities of these molecules, including targeting either the PI3K/PDK1/AKT pathway, receptor tyrosine kinases (RTKs) and/or lipid kinases such as PI3′K. Indeed, side-by-side comparison of PDK1-inhibitor-treated PC3 and LNCap cells confirmed that p-AKT^Thr308^ is not a universal PD biomarker for PDK1 kinase inhibitor development whereas p-RSK^Ser211^ appears to be consistently inhibited across cell lines (Figure 2E). The observation that PDK1 activity does not appear to be rate-limiting for phosphorylation of AKT^Thr308^ in LNCap cells would be consistent with the extensive pathway cross talk and compensatory mechanisms (e.g. negative/positive stimuli and feedback loops) that have been reported for the PI3K-pathway (Figure 2F). Our observation also supports recent genetic PDK1 knock-down studies in PTEN deficient mouse tumor models showing only marginal effects on Thr308 phosphorylation [33]. Consequently, because inhibition of p-AKT^Thr308^ does not directly correlate with the degree of PDK1 target inhibition in cells we set out to identify novel drug-regulated phosphorylation sites on PDK1 as potential candidates for direct read-out of drug-target engagement and inhibition of PDK1.

**Figure 3:**
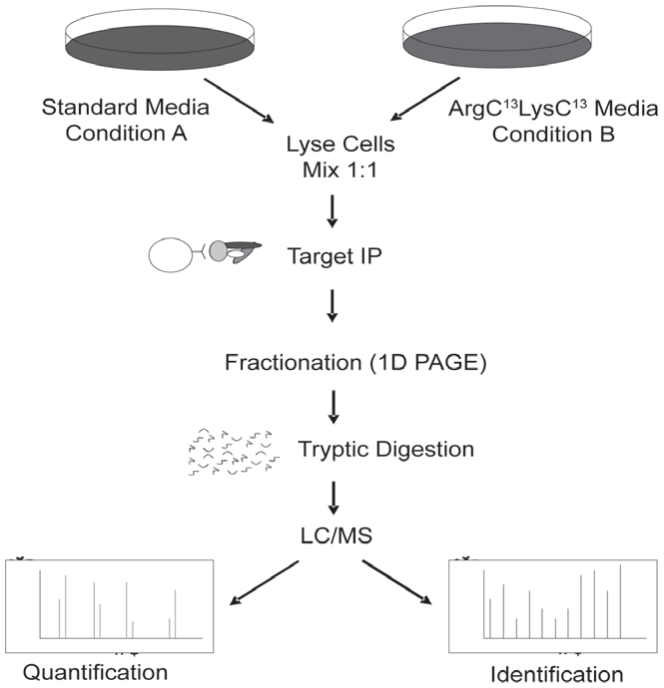
Schematic of stable isotope labeling with amino acid in cell culture and immuno-affinity precipitation for MS analysis (SILAC-IAP-MS). T293 cells grown in either light (^12^C-Arg, ^12^C-Lys) or heavy (^13^C-Arg, ^13^C-Lys) medium were lysed and combined at a 1∶1 ratio based on total protein quantity. PDK1 was immunoprecipitated and analyzed by mass spectrometry. MS raw data were quantified by the Elucidator software 3.5 and confirmed by manual validation as described in the Materials and Methods.

**Figure 4:**
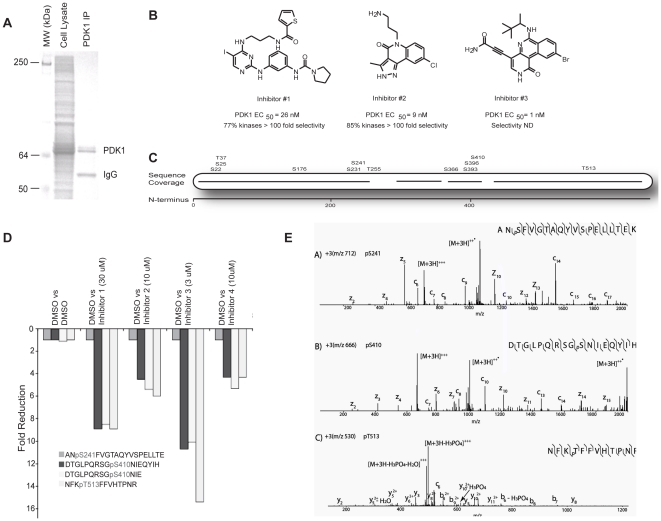
Relative quantification of hPDK1 phosphorylation from 293T cells treated with ATP competitive PDK1 inhibitors using SILAC-IAP-MS. **A**) SDS-PAGE and isolation of the PDK1 protein band for LC-MS/MS analysis. **B**) PDK1 tool compounds representing three structurally distinct chemical classes. PDK1 enzymatic activity is shown (EC_50_). **C**) Mapping of phosphorylation sites of immunoprecipitated PDK1 from 293T cells identifies 12 Ser/Thr phosphorylation sites with ∼95% sequence coverage. **D**) Relative quantification by SILAC-IAP-MS showed a greater than 4 fold reduction of two phosphorylation sites (pS410, and pT513) in samples treated with PDK1 inhibitors compared to samples treated with DMSO. In contrast, no change in phosphorylation was detected in the self-to-self control sample where 293T cells grown in heavy and light media were both treated with DMSO prior to mixing. **E**) MS/MS spectra for pS241 (ANpSFVGTAQYVSPELLTEK), pS410 (DTGLPQRSGpSNIEQYIH), and pT513 (NFKpTFFVHTPNR) containing peptides.

In a more unbiased effort to delineate the effects of ATP-competitive PDK1 inhibitors on the kinase itself, we performed quantitative SILAC mass spectrometric (MS) phosphorylation site analysis on immunoprecipitated PDK1 from cells treated with 4 structurally diverse PDK1 inhibitors for 24 hrs according to the workflow shown in Figure 3. The change of a particular phosphorylation site of interest was derived by comparing the signal intensity of the isotopically labeled “heavy” and radioisotope neutral “light” peptide pair, which was further normalized against a vehicle-to-vehicle control [34]. While desirable, we were unsuccessful at immunopurifying endogenous PDK1 protein from cells in sufficient quantities for subsequent MS analysis. Therefore, we over expressed MYC-tagged PDK1 in 293T cells as further described in Materials and Methods. We, subsequently, immunopurified PDK1 from cells that were drug treated (grown in ‘heavy’ isotope-labeled media), and DMSO treated (grown in ‘light’ media) (Figures 4A & 4B). In-gel proteolytic digestion coupled with mass spectrometric analysis identified 12 phosphorylation sites with ∼95% sequence coverage of full length PDK1 (Figure 4C). Interestingly, amongst these, three phosphorylated peptides which harbor the PDK1 amino acid residues Ser410 and Thr513 showed a greater than 4-fold reduction in phosphorylation when treated with the four structurally diverse ATP-competitive PDK1 inhibitors compared to vehicle control (Figure 4D). In contrast, peptides containing phosphorylated Ser241 were not modulated under any of the treatment conditions tested, consistent with Western blotting analysis (Figure 4D and data not shown). Notably, two distinct phosphopeptides harboring pPDK1^Ser410^ exhibited the same reduction in phosphorylation by each of the small molecule inhibitors (Figure 4D). The corresponding MS/MS spectra for p-Ser241 (ANpSFVGTAQYVSPELLTEK), p-Ser410 (DTGLPQRSGpSNIEQYIH), and p-Thr513 (NFKpTFFVHTPNR) are shown in Figure 4E. These results support the use of immunoaffinity precipitation (IAP–MS) to identify and quantify drug-specific phosphorylation changes as a means to measure drug target engagement in cells.

## Discussion

**Figure 5:**
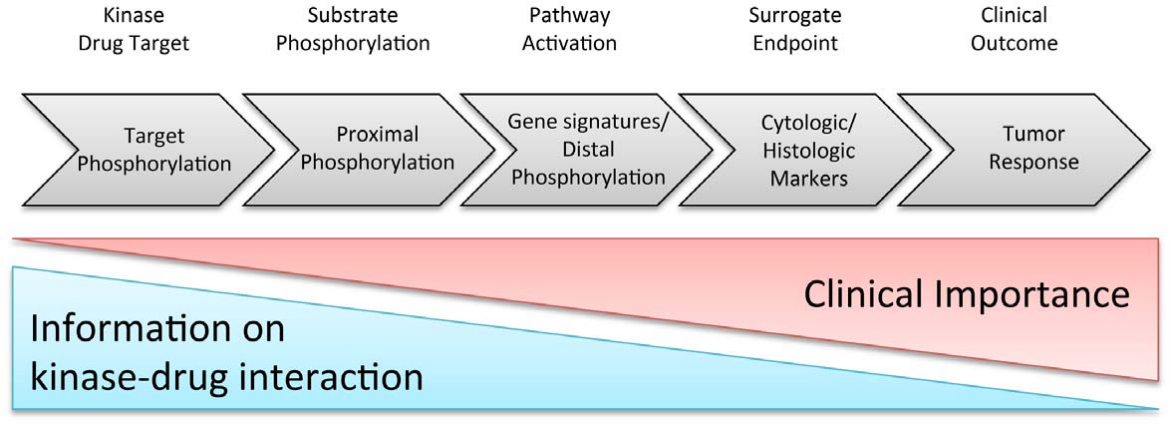
The pharmacological biomarker audit trail. Biomarker categories demonstrate the relationship between the specificity of target engagement biomarkers (i.e. proximal biomarkers) and distal disease-related outcome biomarkers. Direct target engagement markers allow lead optimization of compounds at the kinase target level.

The human genome encodes a little more than 500 protein kinases and an estimated twenty percent of drugs currently in development target this enzyme class [24]. To successfully identify, develop, and utilize biomarkers for novel drug targets a question-based pharmacological audit-trail has been proposed [35], [10]. The answers are provided by fit-for-purpose biomarkers gating the development of the molecule (Figure 5). Arguably, target engagement and downstream PD markers are to be incorporated early on in the process as they collectively inform on biochemical target modulation with consequent downstream pathway effects. With an in depth understanding of drug efficacy in terms of target engagement, pharmacokinetic and pharmodynamic (PK-PD) relationships can be established in preclinical animal models and ultimately inform on both drug dose and schedule in human clinical studies.

Examples already exist for RTK inhibitors where inhibition of T-loop autophosphorylation of the drug target provides a direct readout for the degree of drug target inhibition. For example, Dasatinib is a multi-targeted inhibitor of BCR-Abl and SRC family kinases, which has clinical activity in CML. Modulation of BCR-ABL phosphorylation in combination with phosphorylation of its downstream substrate CRKL has been successfully used to inform clinical decision-making [36]. Following a single oral administration of Dasatinib at preclinical efficacious doses, mathematical modeling of PK-PD relationships was used to predict the human plasma concentration needed to achieve 90% target inhibition (IC_90_) [36]. If these results are applicable to kinase targets in general, drug-specific phosphorylation modulations of the kinase itself can provide for direct target engagement markers. When complemented with knowledge of drug plasma and/or tissue concentration, desired biological effects, such as cell cycle arrest or apoptosis in cancer cells, can be used as evidence for the molecular interaction of the drug with the target *in vivo* and hence inform about true drug target exposure. Such combined information has far-reaching potential, and can provide evidence whether a molecule fails clinical proof-of-concept (POC) for efficacy in clinical trials due to the biological mechanism or simply due to lack of target modulation. It is important to emphasize that target engagement biomarkers, while defining the molecular effects of the drug on the purported target, may not necessarily have any correlation with the desired clinical effects (Figure 5). Knowledge about good drug target exposure yet no desirable clinical or biochemical efficacy might warrant further investigation into the biological role of the target in disease biology

In this study, we present a quantitative phosphoproteomics based approach to discover direct target engagement biomarkers for Ser/Thr protein kinase drug discovery focusing on the PI3′K pathway target PDK1 as an example (Figure 2). The discovery of novel drug-regulated phosphorylation sites on this target produces evidence directly correlating the target engagement/PD biomarker to the pathway inhibition (Figure 4). We identified p-Ser410 and p-Thr513 to be regulated by classical, type I ATP competitive PDK1 inhibitors in a dose-dependent correlation, while p-Ser241 was not modulated at all. Very limited information exists for the regulatory effect of these phosphorylation sites on PDK1 activity with only 2 reports to date discussing the importance of p-Ser410 [27] and of p-Thr513 [37] phosphorylation for the PDK1 enzyme activity.

To demonstrate that the herein described approach is more universally applicable (Figure 3) we employed the same strategy to identify drug-regulated target engagement phosphorylation markers for two additional protein kinases, the serine/threonine protein kinase MARK3 as well as a RTK, c-Met. We found that transiently transfected MARK3 exhibited substantial phosphorylation on 22 sites based on our proteomics analysis, with ∼95% of sequence coverage (Figure S1). Amongst these 22 phosphorylation sites, 17 had signal intensities that were quantifiable with nine phosphorylation sites reproducibly decreased by two structurally diverse inhibitors of MARK3 (Figure S1). Specifically, signal intensities of peptides containing phosphorylation at Ser390/391, Ser400, and Ser469 showed a dose-dependent decrease in response to both compounds. In contrast, p-Ser19, p-Ser598/601 and p-Ser583/587 were strongly inhibited by only one of the compounds. Similarly, the herein described methodology has proven equally successful for RTKs [16]. Differential mass-spectrometry of endogenously immunoprecipitated c-Met from GTL-16 cells shows that MK-2461, a highly specific c-Met inhibitor, strongly modulates not only the T-loop activation loop (Y1230/Y1234/Y1235), but also phosphorylation sites that are within the COOH-terminal docking site (Y1349 and Y1365) (Figure S2 and Table S1). These examples demonstrate that our proposed method will enable differentiation of small molecules developed against the same targets. The variable effects of these drugs on the target are likely due to different binding characteristics and/or binding sites, resulting in different allosteric conformations with variable effects on kinase autophosphorylation. Another reason could be different off-target -effects resulting in variable indirect effects on target phosphorylation due to the complex network of interactions inside a cell.

A key question is how such novel drug target engagement-regulated phosphorylation sites can be translated into clinical, practical usage. The answer to this question depends in part where to measure and what analytical assays are to be employed. The strength of the herein laid out approach is to broadly identify and quantify effects of molecules on the phosphorylation status without *a priori* knowledge. However, we note that the identification of candidate target engagement markers is performed *in vitro*, and importance is initially inferred from corresponding potency data. The answer to the question ‘which phosphorylation sites are modulated *in vivo*’ requires access actual diseased human tissue and appropriate tools. We and others are advocating a pre and post-dose biopsy driven trial design in where pathway and target modulation is directly assessed in the target tissue of interest [5], [38]. In cases where specific antibody reagents are available, biochemical and/or immunohistochemical (IHC) assays can be established for use in the clinic, or if needed, phosphorylation specific antibodies must be generated. However, as this study has illustrated, many of the drug-regulated sites are either novel, and their immunogenicity may be low. Given the fact that mass spectrometry is at the core of the described approach and essential for the discovery of these biomarkers, it is plausible that the future development of highly sensitive, robust, selective, and absolute quantitative MS based assays could become an attractive alternative approach when antibody reagents are not available or not easily generated [39]–[42]. While conceptionally straightforward, significant improvements of phospho-MS based assays, in the form of increasing sensitivity in MS instrumentations and robustness of biochemical isolation from minute amounts of clinical materials, are yet to be realized.

## Methods

### Materials

LNCap, PC3, and 293FT cells were purchased from American Tissue Type Collection. All antibodies were used at a concentration of 1∶1000 and purchased from Cell Signaling Technology, Inc. Metablolic SILAC labeling kits were purchased from Invitrogen. All other reagents were purchased from their respective manufactures as described below.

### Cell culture and SILAC labeling

T293 cells were grown for seven passages in DMEM media containing either normal-isotope lysine, arginine (‘light medium’) or ^13^C_6_ containing lysine and arginine (‘heavy medium’) in high glucose, L-glutamine, 110 mg/L sodium pyruvate and 10% FBS. Myc-tag PDK1 (pCMV5-PDK1-wt) was transiently transfected into 293FT cells (5×10^6^ cells per 10 cm petri dish) using standard 293FT LV packaging transfection protocols (i.e. lipofectamine in OPTI-MEM). Kinase inhibitors or control (DMSO) were added 18 hours prior to cell lysis using immunoprecipitation buffer (50 mM Tris-HCL, pH 7.4, 150 mM NaCl, 1% NP-40, 1 mM EDTA, protease inhibitor tablet (Roche), 1 mM b-Glycerolphosphate, 1 mM Na_3_VO_4_, 2.5 mM Sodium pyrophosphate). ‘Heavy’ and ‘light’ isotope-labeled samples were mixed 1∶1 based on the protein concentration measurement using standard BCA protocol. 250 µl lysate was pre-cleared by adding 30 µl of a 50% Protein-A agarose slurry for ∼2 hours and PDK1 was immunoprecipitated via tag-specific pull down (anti-c-MYC antibody) and subjected to SDS-PAGE and stained with Coomassie blue. The identical procedure was also carried out for a 1;1 mix of cells grown in ‘heavy’ and ‘light’ isotope without compound treatment as an internal control.

### Western Blot analysis

Western blotting was performed for 2 h using a BioRad SemiDry apparatus with Immobilon-P PVDF membrane (Millipore), at a constant voltage of 25 V and 10A/10 cm2. Chemiluminescent detection was carried out using CDP Star™ (Tropix, Bedford, MA, USA) per recommendations by the manufacturer.

### Luminex and In-Cell assays for p90RSK^Ser221^ and pAKT^Thr308^

Development of biochemical assays to report on phospho-p90RSK^Ser221^ and phospho-AKT^Thr308^ were previously reported and performed as described [30], [31].

### Biochemical processing and mass spectrometry

Excised protein bands were reduced, alkylated and digested with Trypsin (Promega), GluC (Roche) and AspN (Roche) overnight at the room temperature. The resulting peptides were analyzed by a reverse-phase nano-liquid chromatography system (1100 Series HPLC system; Agilent Technologies) online-coupled with a LTQ Orbitrap hybrid mass spectrometer with electron transfer dissociation (LTQ Orbitrap XL ETD) (ThermoFisher Scientific). Briefly, an aliquot of the digested sample was loaded onto a micro-capillary liquid chromatography column (50 µm i.d.×25 cm packed with Magic C18). Bound peptides were eluted from the column via a 75 min solvent gradient program. Eluted peptides were continuously converted to multiply charged peptide ions by electrospray ionization and introduced into the vacuum system of a LTQ Orbitrap XL ETD. High resolution mass spectra were acquired at a rate of 1 spectrum per second. These spectra recorded the intensity, retention time, and accurate mass-to-charge ratio for the discrete peptide ions in each sample. MS/MS spectra that were simultaneously acquired were used to provide amino acid sequence information for selected peptide ions.

### Data analysis

Acquired MS data were uploaded to the Elucidator data analysis system (Rosetta Biosoftware, version 3.5) as previously described [5], [43] for feature extraction, quantitative analysis and searched against a human PDK1 database obtained from National Center for Biotechnology Information (NCBI) http://pubchem.ncbi.nlm.nih.gov/ using SEQUEST. Parameters for the searches included the following: Trypsin, AspN, GluC for enzyme specificity respectively; static modifications of 57.02 Da on Cys (Carbamidomethylation); differential modification of 79.9 Da on Ser, Thr, and Tyr (phosphorylation) and 15.99 Da on Met (oxidation). The precursor ion mass tolerance was set as 50 ppm and the fragment-ion mass tolerance was set as +/−0.8 Da Mono/Mono. Each MS/MS spectra exhibiting possible phosphorylation were manually validated. ‘Light’ and ‘heavy’ SILAC labeled peptide pairs are identified based on *m*/*z* and the retention time of the features contained in the data set. The ratio of the identified peptide pairs were then derived based on the ratio of their corresponding areas under the ion chromatographic curves.

## Supporting Information

Figure S1 **Relative quantification of MARK3 phosphorylation by SILAC-IAP-MS.** **A**) MARK3 targeting inhibitors. **B**) Relative quantification by SILAC-IAP-MS showed the signal intensity of three phosphorylation sites (pS469, pS390/391 and pS400) were dose-dependently reduced in response to both inhibitor 5 and 6.
(TIF)

Figure S2 **Relative quantification of c-Met phosphorylation by SILAC-IAP-MS.** **A**) Fold changes for c-Met phosphopeptides treated with MK-2461 treatment vs. DMSO (red columns) were obtained as described in materials and methods. **B**) Reconstructed ion chromatogram and **C**) high resolution full MS spectra of both light (black) and heavy (red) peptides ISAIFSTFIGEHpYVHVNATpYVNVK (Y1349/Y1356) respectively.
(TIF)

Table S1 **Relative quantification of c-Met phosphorylation by SILAC-IAP-MS.** DMSO and IC90 refer to treating cells either using DMSO or MK-2461 at IC90 concentration, (L) and (H) refer to light and heavy isotope media.
(DOC)
