# Supplementary figures and images for: Identification of Direct Target Engagement Biomarkers for Kinase-Targeted Therapeutics
Source: PLoS One. 2011 Oct 24;6(10):e26459. doi: 10.1371/journal.pone.0026459 (PMC3200335; doi:10.1371/journal.pone.0026459)

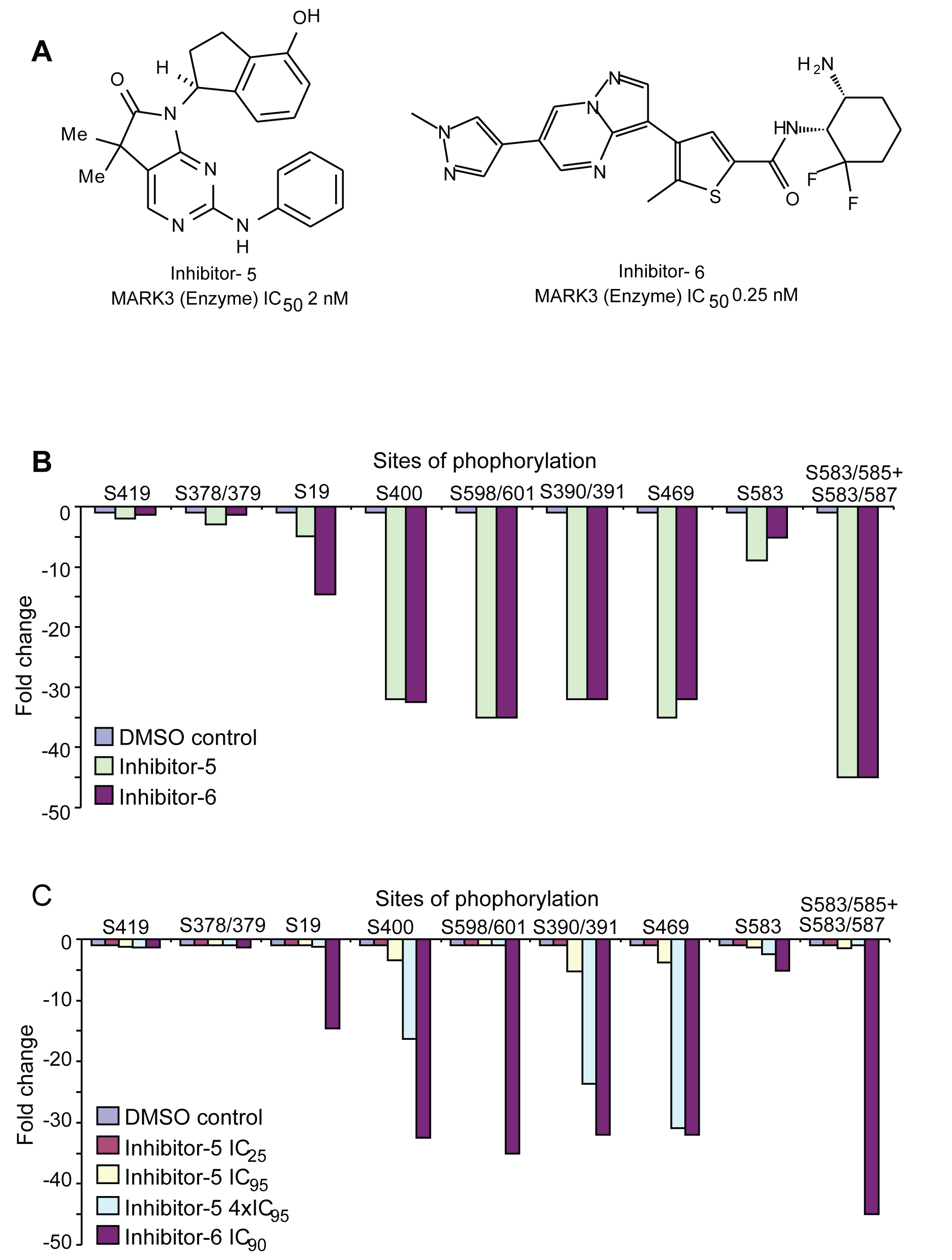

Supplement: Figure S1 — Relative quantification of MARK3 phosphorylation by SILAC-IAP-MS. A) MARK3 targeting inhibitors. B) Relative quantification by SILAC-IAP-MS showed the signal intensity of three phosphorylation sites (pS469, pS390/391 and pS400) were dose-dependently reduced in response to both inhibitor 5 and 6. (TIF) [file pone.0026459.s001.tif]

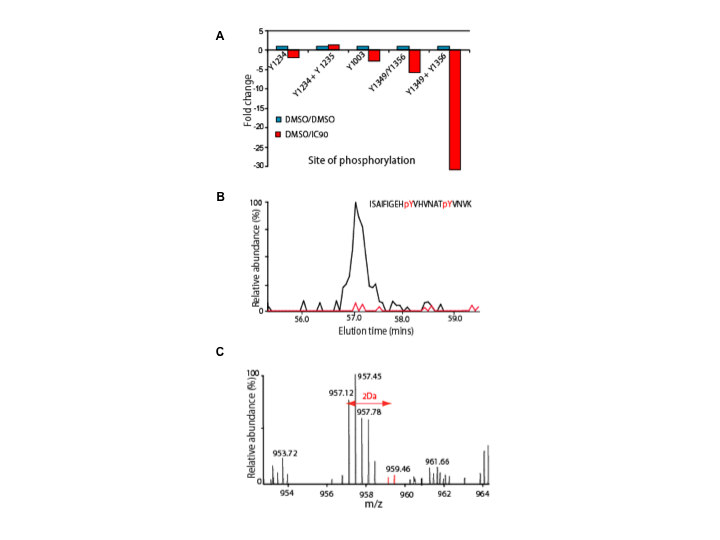

Supplement: Figure S2 — Relative quantification of c-Met phosphorylation by SILAC-IAP-MS. A) Fold changes for c-Met phosphopeptides treated with MK-2461 treatment vs. DMSO (red columns) were obtained as described in materials and methods. B) Reconstructed ion chromatogram and C) high resolution full MS spectra of both light (black) and heavy (red) peptides ISAIFSTFIGEHpYVHVNATpYVNVK (Y1349/Y1356) respectively. (TIF) [file pone.0026459.s002.tif]
