# Supplementary material for: Identification of Direct Target Engagement Biomarkers for Kinase-Targeted Therapeutics
Source: PLoS One. 2011 Oct 24;6(10):e26459. doi: 10.1371/journal.pone.0026459 (PMC3200335; doi:10.1371/journal.pone.0026459)
Supplement: Table S1 — Relative quantification of c-Met phosphorylation by SILAC-IAP-MS. DMSO and IC90 refer to treating cells either using DMSO or MK-2461 at IC90 concentration, (L) and (H) refer to light and heavy isotope media. (DOC) [file pone.0026459.s003.doc]

| **Site** | **Detected peptide sequence** | **Fold changes** | **DMSO (L)** | **IC90**  **(H)** |
| --- | --- | --- | --- | --- |
| Y1003 | **SVSPTTEMVSNESVDpYR** | -2.8 | 14% | 5% |
| Y1234 | **DMYDKEpYYSVHNK** | -1.9 | 30% | 16% |
| Y1234 + Y1235 | **DMYDKEpYpYSVHNK** | 1.3 | 12% | 15% |
| Y1349/ Y1356 | **ISAIFSTFIGEHpYVHVNATYVNVK**  **or**  **ISAIFSTFIGEHYVHVNATpYVNVK** | -5.8 | 11% | 2% |
| Y1349 + Y1356 | **ISAIFSTFIGEHpYVHVNATpYVNVK** | -50 | 2% | <0.04% |
